# Abnormal theta and alpha oscillations in children and adolescents with first-episode psychosis and clinical high-risk psychosis

**DOI:** 10.1192/bjo.2024.32

**Published:** 2024-03-22

**Authors:** Yaru Zhang, Tingyu Yang, Yuqiong He, Fanchao Meng, Kun Zhang, Xingyue Jin, Xilong Cui, Xuerong Luo

**Affiliations:** Department of Psychiatry, National Clinical Research Center for Mental Disorders and National Center for Mental Disorders, The Second Xiangya Hospital of Central South University, China; National Clinical Research Center for Mental Disorders and Beijing Key Laboratory of Mental Disorders, Beijing Anding Hospital, Capital Medical University, China; and Advanced Innovation Center for Human Brain Protection, Capital Medical University, China; Department of Child and Adolescent Psychiatry, Suzhou Guangji Hospital, China; and Department of Child and Adolescent Psychiatry, Affiliated Guangji Hospital of Soochow University, China

**Keywords:** Children and adolescents, electroencephalogram, N2, oscillations, psychosis

## Abstract

**Background:**

Cognitive control deficits are one of the main symptoms of psychosis. The basic neural oscillation patterns associated with cognitive control are already present in early adolescence. However, as previous studies have focused on adults with psychosis, it is unclear whether neurobiological impairments in cognitive control are present in children and adolescents with first-episode psychosis (FEP) or clinical high-risk (CHR) state for psychosis.

**Aims:**

To explore the deficits of electroencephalogram related to cognitive control tasks in children and adolescents with FEP and CHR.

**Method:**

Electroencephalogram was recorded in untreated 48 patients with FEP, 24 patients with CHR and 42 healthy controls aged 10–17 years, while performing the visual oddball task. The N2 amplitude, theta and alpha oscillations were then analysed and compared between groups.

**Results:**

There was no significant group difference in N2 amplitude (*P* = 0.099). All groups showed increased theta and alpha oscillations relative to baseline before the stimulus in the frontal, central, left fronto-central and right fronto-central areas. These changes differed significantly between groups, with the FEP group showing significantly smaller theta (*P* < 0.001) and alpha (*P* < 0.01) oscillation than healthy controls. Theta and alpha oscillations in the CHR group did not differ significantly from the FEP group and healthy controls.

**Conclusions:**

These results suggest that neural damage has already occurred in the early stage of psychosis, and that abnormal rhythmic activity of neurons may constitute the pathophysiological mechanism of cognitive dysfunction related to early-onset psychosis.

Psychosis is a severe, chronic mental illness of unknown aetiology that typically begins in late adolescence or early adulthood. Onset before 18 years of age is termed early-onset psychosis, and is commonly characterised by atypical psychotic symptoms, highly implicit cognitive impairment, more severe disease forms and poor prognosis.^1^ Cognitive control deficits are one of the main symptoms of psychosis.^2^ Cognitive control is a goal-oriented, flexible and timely process of adjusting intentions and actions,^3,4^ including inhibitory control, working memory updating and cognitive flexibility. Cognitive control develops throughout childhood until near maturity in adolescence. Previous studies have shown that cognitive control deficits in psychosis are associated with decreased prefrontal cortex and frontal parietal network involvement.^5^ Understanding the neural mechanisms leading to cognitive control deficits is thus a key step for early intervention and the development of targeted therapies for psychosis.

## Electroencephalogram changes in psychosis with cognitive control deficits

In recent years, electroencephalogram has been used to examine the neural mechanisms of cognitive control.^6,7^ N2 is a negative event-related potential (ERP) component that appears approximately 200–300 ms after stimulation, usually in the central prefrontal region.^3^ N2 is thought to reflect top-down cognitive processes, which are likely attributed to the perception of novel stimuli and the orientation of visual attention in cognitive control regulation.^3^ Reduced N2 amplitude was found in patients with a variety of psychiatric disorders, including psychosis, which may reflect cognitive control dysfunction in patients with psychosis.^8^

In recent years, increasing studies have explored the local and distributed neural circuit mechanisms underlying ERP impairment through time-frequency analysis.^9^ Large-scale neuronal integration is formed by synchronisation or de-synchronisation of neurons in different frequency bands, also termed event-related synchronisation (ERS)/event-related de-synchronisation and neural oscillation, which represent increases or decreases in the power of a specific frequency band when an event occurs. This process is related to the neural mechanisms of cognitive activity,^10^ and allows rapid and efficient communication between neurons in the brain. Theta band (4–7 Hz) oscillations are involved in cognitive control and top-down processes,^11^ such as the encoding of working memory.^12^ Alpha band (8–13 Hz) oscillations are associated with the distribution of attention,^13^ and reflect processes related to inhibitory functions of the brain.^14^

Abnormal theta oscillations have been reported in patients with psychosis. For example, one study used the Stroop word interference task and found that patients with psychosis have a significantly smaller increase in theta oscillations in the left frontal and parietal regions than controls.^15^ Another study of event-related spectral perturbations of misprediction tasks found that theta oscillations of the prefrontal lobe were significantly reduced in patients with psychosis compared with controls.^16^ Furthermore, patients with psychosis show abnormal alpha oscillations. For example, during a self-referential task, people with schizophrenia showed abnormally decreased alpha oscillations in the prefrontal, parietal and occipital regions 100–300 ms after stimulation.^17^ Attention-related regulation of alpha activity is also significantly impaired during visual processing tasks in people with schizophrenia.^18^

## The current study

There is an emerging idea that the basic patterns based on neural oscillations associated with cognitive control are already present in early adolescence.^19^ However, as previous studies on cognitive control in psychosis have focused on adults with a chronic disease course, it is unclear whether neurobiological impairments in cognitive control occur in children and adolescents with first-episode psychosis (FEP) or earlier stages of the disease, i.e. clinical high-risk (CHR) state for psychosis, which is characterised by attenuated symptoms of psychosis, impairment of social function and a higher risk of conversion to psychosis spectrum disorder (approximately 30% within 2 years).^20^ To address this research gap, we investigated N2 amplitude characteristics in unmedicated children and adolescents with FEP or CHR, specifically the characteristics of theta and alpha oscillations, to better understand the neuroelectrophysiological basis of cognitive control impairment in early-onset psychosis.

## Method

### Participants

From August 2018 to April 2021, 48 patients with FEP (aged 12–17 years) and 24 patients with CHR (aged 10–17 years) were recruited from the psychiatric clinic and ward of the Second Xiangya Hospital in Hunan Province, China. Patients were diagnosed according to the DSM-5^21^ criteria for FEP and Structured Interview for Psychosis-Risk Syndromes (SIPS)^22^ criteria for CHR. SIPS has been used to identify different types of CHR, which classifies CHR into three types: attenuated positive symptom syndrome, transient intermittent psychotic syndrome and genetic risk and degenerative syndrome.^22^Additionally, 42 healthy controls aged 10–17 years were recruited by advertising in schools. Psychiatric symptoms in all patients were assessed by a trained psychiatrist with the Positive and Negative Syndrome Scale (PANSS).^23^ None of the patients received antipsychotic drugs or other therapy before participating in this study. All participants were right-handed and had normal or corrected normal vision. The exclusion criteria for patients included a history of other mental diseases, any central nervous system disease, intellectual disability, serious physical diseases, alcohol or drug misuse, or inability to cooperate with the study protocol. For healthy controls, the exclusion criteria were any history of mental or neurological disorders (confirmed by an interview with a trained psychiatrist using the Kiddie Schedule for Affective Disorders and Psychosis – Present and Lifetime Version),^24^ intellectual disability, alcohol or drug misuse, or family history of mental illness within three generations.

The authors assert that all procedures contributing to this work comply with the ethical standards of the relevant national and institutional committees on human experimentation and with the Helsinki Declaration of 1975, as revised in 2008. All procedures involving human patients were approved by the Second Xiangya Hospital of Central South University, Changsha, China (approval number: 2018030). All participants and their parents/guardians were voluntary and provided written consent.

### Experimental paradigm

The experiment was conducted by a professionally trained psychiatrist in a quiet, soundproof electroencephalogram room, maintained at a constant temperature. Before the beginning of the experiment, the researchers ensured that the participants fully understood the experimental process. Participants were seated 60 cm away from the computer display and instructed to look directly at the centre of the screen. The visual oddball paradigm was designed and presented with E-prime 2.0 software for Windows (Psychology Software Tools Inc., Pittsburgh, PA, USA; see https://pstnet.com/products/e-prime/). Participants were instructed to press the ‘F’ or ‘J’ key in response to the random standard stimulus (letter ‘W’, probability of 80%) and target stimulus (letter ‘m’, probability of 20%), respectively. The paradigm was divided into three blocks: the first block comprised 15 practice trials, and the next two blocks comprised the formal experiment of 125 trials each. At the beginning of the experiment, a blank screen was presented for 500–1500 ms, followed by a black ‘+’ symbol for 500 ms to remind the participants to concentrate, and then 2000 ms of stimulation.

### Electroencephalogram preprocessing and ERP recording

Electroencephalogram data were preprocessed using EEGLAB in the MATLAB platform for Windows (R2013b, The MathWorks, Inc., Natick, MA, USA; see https://www.mathworks.com). The sampling rate of electroencephalogram data was first reduced to 500 Hz, and then filtered (high-pass filtering >0.1 Hz, low-pass filtering <30 Hz and notch filtering from 48 to 52 Hz). Segmentation was performed from 500 ms before the stimulus to 800 ms after the stimulus, and only corrected trials were kept. Next, we eliminated bad segments and interpolated bad leads, using the spherical interpolation method to interpolate abnormal leads. Independent component analysis was used to remove artifacts such as eye movements, electrical components and oscillation frequency interference. Electrodes at the bilateral mastoid (TP9/TP10) were used as reference electrodes. Finally, extreme values (voltage amplitude exceeding ±100 μV) were removed. All segments were superimposed and averaged to obtain the ERP waveform. The time window for N2 was determined from a previous study^25^ and inspection of the ERP grand averages. In this way, the N2 amplitude was defined as the average amplitude between 200 and 350 ms after the stimulus. N2 was detected in the frontal region where the N2 amplitude is greatest. The following four regions of interest (ROIs) were selected for analysis: ROI1 (F1, Fz, F2), ROI2 (C1, Cz, C2), ROI3 (FC1, FC3, FC5) and ROI4 (FC2, FC4, FC6). These ROIs correspond to the frontal, central, left fronto-central and right fronto-central brain regions, respectively.

### Time-frequency analysis

Theta band (4–7 Hz) and alpha band (8–13 Hz) oscillations were only analysed in correct trials. Theta and alpha oscillations were calculated by using MATLAB to apply the short-time Fourier transform to individual trials, and electroencephalogram data were converted into the time-frequency domain. Theta and alpha oscillations were measured for each participant, in ROI1, ROI2, ROI3 and ROI4. The frequency range was set to 0.1–30 Hz in 0.5 Hz steps. As the time-frequency transformation results were not perfectly accurate at the beginning and end of trials, 350–150 ms before the stimulus was used as the baseline range. The time-frequency plots after baseline correction were obtained by subtracting the average oscillations of the baseline period from the power of each time-frequency point after stimulation. We calculated the average theta and alpha oscillations in the time window of 200–350 ms after the start of stimulation. The time-frequency characteristics of electroencephalogram were visualised by time-frequency analysis and topographic maps.

### Statistical analysis

Statistical analysis was performed with SPSS version 22.0 (IBM, Chicago, Illinois, USA). Quantitative variables are expressed as the mean ± s.d. and median (third quartile, first quartile), whereas categorical variables are expressed as the frequency (*n*) and composition ratio. Continuous data were compared between the three groups, using one-way analysis of variance (ANOVA). Chi-squared test was used for comparison of categorical data. The Kruskal–Wallis rank-sum test was used to compare data that did not conform to normal distribution or homogeneity of variance. Three-factor repeated measure ANOVA was used to examine the effects group (FEP, CHR, healthy controls) × ROI (ROI1, ROI2, ROI3, ROI4) × stimulus type (standard, target) on N2 amplitude, theta oscillations and alpha oscillations. Greenhouse–Geisser correction for sphericity was applied. Bonferroni correction was applied for pairwise comparisons.

## Results

### Participant characteristics

Table 1 presents the general demographic data and clinical characteristics of the FEP, CHR and healthy control groups. For the FEP and CHR groups, the duration of illness and PANSS score were provided. For the CHR group, the Scale of Psychosis-Risk Syndromes Positive, Negative, Disorganized and General symptom total scores were also provided. *Post hoc* analyses revealed no significant differences between any two groups (*P* > 0.05), despite the Kruskal-Wallis rank-sum test indicating an age difference between the three groups (*P* = 0.030). Age was therefore excluded as a covariate in the analyses that followed. There was no significant difference in gender composition among the three groups (*P* = 0.477).

**Table 1:** General demographic data and clinical characteristics of the three groups

Effect	FEP (*n* = 48)	CHR (*n* = 24)	Healthy controls (*n* = 42)	F/χ^2^	*P-*value
Age (years)	15.06 ± 1.69	13.95 ± 1.97	14.23 ± 2.18	7.019	0.030^a^
Gender, % (*n*)				1.479	0.477
Male	39.60% (19)	45.80% (11)	52.40% (22)		
Female	60.40% (29)	54.20% (13)	47.60% (20)		
Duration of illness (months)	4 (7.75, 2.25)	4 (8.00, 2.00)	−	0.167	0.868
PANSS score					
Positive symptoms	20.05 ± 5.14	15.13 ± 4.24	−	3.285	0.002
Negative symptoms	20.26 ± 6.93	16.60 ± 5.61	−	1.821	0.074
General pathology symptoms	41.58 ± 8.90	27.87 ± 6.58	−	5.398	<0.001
SOPS score					
Positive	−	10.93 ± 2.46	−		
Negative	−	11.40 ± 2.59	−		
Disorganization	−	9.80 ± 2.21	−		
General	−	7.40 ± 0.99	−		
Correct response rates	93.80% ± 8.89%	95.54% ± 3.17%	96.58% ± 1.87%	2.412	0.094

Data are represented as the mean ± s.d. or median (third quartile, first quartile). FEP, first-episode psychosis; CHR, clinical high risk; PANSS, Positive and Negative Syndrome Scale; SOPS, Scale of Psychosis-Risk Syndromes.

a. *Post hoc* tests showed no statistically significant difference.

### N2 amplitude

As shown in Table 2, the group × region × stimulus interaction effect was not statistically significant for N2 amplitude (F(6,220) = 1.393, *P* = 0.219). However, the main effect of stimulus type was significant (F(1,111) = 14.922, *P* < 0.001). *Post hoc* tests showed that the N2 amplitude evoked by the target stimulus was significantly greater than that evoked by the standard stimulus. The main effect of brain region (ROI1, ROI2, ROI3, ROI4) was also significant (F(3,109) = 40.500, *P* < 0.001). *Post hoc* tests showed that the N2 amplitude in the frontal region was the highest, being significantly higher than that in the central and other regions (*P* < 0.001). There was no significant main effect of group (*P* = 0.099).

**Table 2:** Effects of different factors on N2 amplitude

Effect	Hypothesis d.f., error d.f.	F/T	*P-*value	Follow-up tests
Group (FEP, CHR, healthy controls)	2, 111	2.358	0.099	
Stimulus (target, standard)	1, 111	14.922	<0.001	target > standard***
Regions (ROI1, ROI2, ROI3, ROI4)	3, 109	40.500	<0.001	ROI1 > ROI3*** = ROI4 > ROI2***
Group × stimulus	2, 111	1.760	0.177	
Group effect for target				
Group effect for standard				
Group × regions	6, 220	3.076	0.007	
Group effect at ROI1				
Group effect at ROI2				
Group effect at ROI3				
Group effect at ROI4				
Group × regions × stimulus	6, 220	1.393	0.219	

FEP, first-episode psychosis; CHR, clinical high risk; ROI, region of interest.

*** *P* < 0.001.

### Theta oscillation

**Fig. 1:**
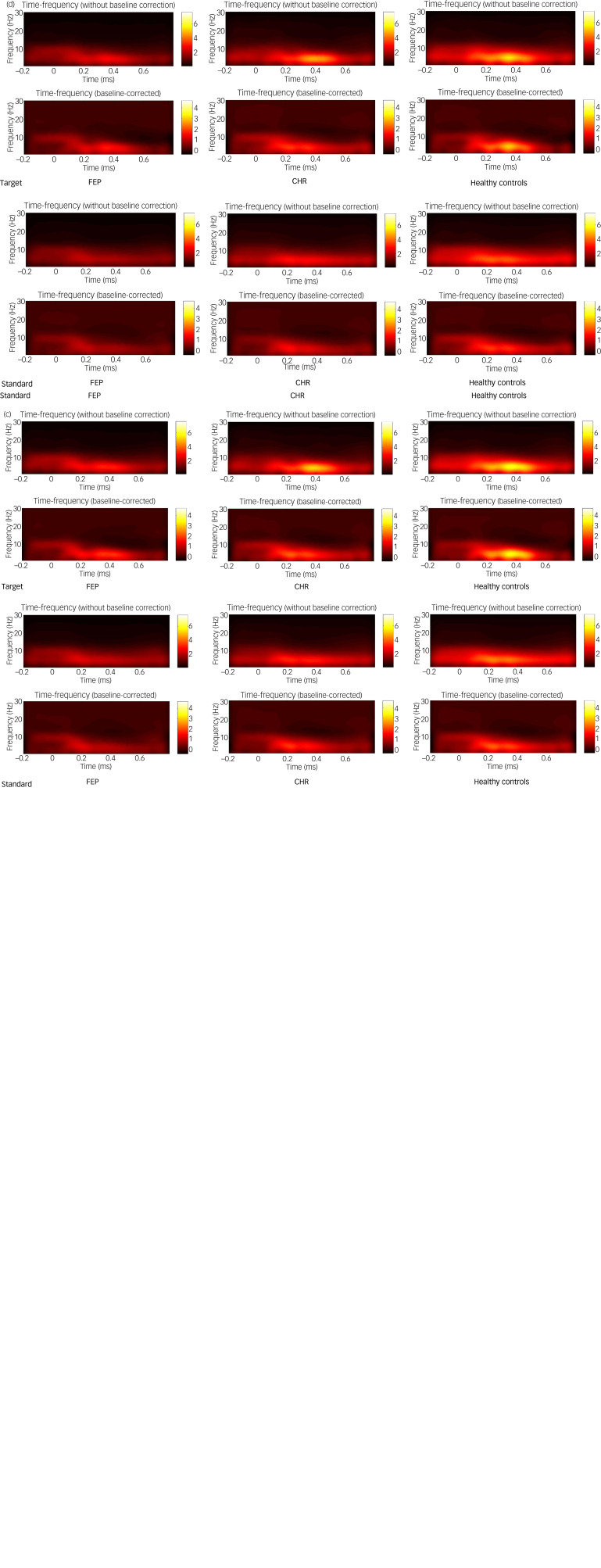
Time-frequency spectrograms illustrating the theta and alpha oscillatory power of the target and standard stimulus in the first-episode psychosis (FEP), clinical high-risk (CHR) and healthy control groups. Windowed Fourier transform was used to transform single-trial electrocortical responses in the time domain into time-frequency distributions to show changes in signal power with time and frequency. Subtraction was used for baseline correction. The *x*-axis represents the time 200 ms before stimulus to 800 ms after stimulus, whereas the *y*-axis represents frequency. Compared with the healthy control group, the FEP group showed significantly decreased theta and alpha oscillatory power in the (a) frontal, (b) central, (c) left fronto-central and (d) right fronto-central region. The colour bar represents the oscillatory power values.

**Fig. 2:**
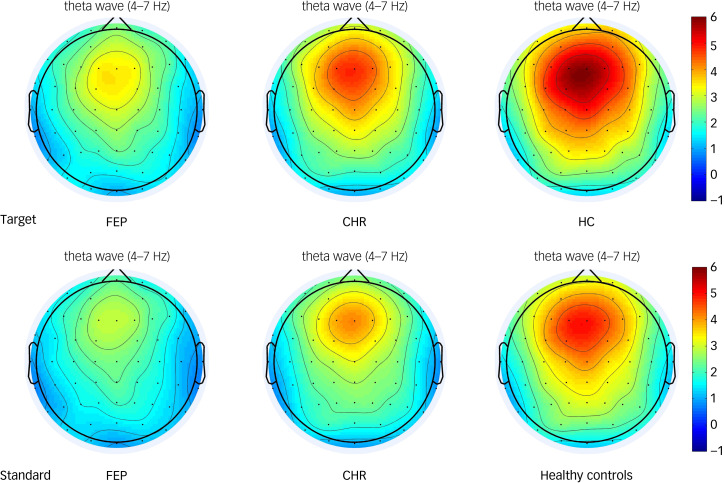
Topographic maps of the theta oscillatory power of the target and standard stimulus in the first-episode psychosis (FEP), clinical high-risk (CHR) and healthy control groups. The colour bar represents the oscillatory power values.

Theta oscillations were increased relative to baseline in the frontal, central, left fronto-central and right fronto-central regions in all three groups (Fig. 1). The group × region × stimulus interaction on theta oscillation was not significant (F(6,220) = 0.864, *P* = 0.522; Table 3). The main effect of stimulus type was significant (F(1,111) = 44.116, *P* < 0.001). *Post hoc* tests showed that the theta oscillation induced by the target stimulus was significantly larger than that induced by the standard stimulus. The main effect of brain region (ROI1, ROI2, ROI3, ROI4) was also significant (F(3,109) = 78.246, *P* < 0.001). *Post hoc* tests showed that theta oscillation in the frontal region was the significantly larger than that in the central region (*P* < 0.001), and that the theta oscillation in the left fronto-central region was significantly larger than that in the right fronto-central region (*P* < 0.001). The main effect of group was significant. *Post hoc* tests showed that theta oscillation in healthy controls was significantly larger than that in the FEP group (*P* < 0.001). However, theta oscillations in the CHR group were not significantly different from those in the healthy control and FEP groups (*P* > 0.05). Topographic maps of the theta oscillations in the three groups to the target and standard stimuli are shown in Fig. 2.

**Table 3:** Effects of different factors on theta oscillation

Effect	Hypothesis d.f., error d.f.	F/T	*P-*value	Follow-up tests
Group (FEP, CHR, healthy controls)	2, 111	9.832	<0.001	Healthy controls > FEP***, Healthy controls > CHR, CHR > FEP
Stimulus (target, standard)	1, 111	44.116	<0.001	target > standard***
Regions (ROI1, ROI2, ROI3, ROI4)	3, 109	78.246	<0.001	ROI1 > ROI2*** = ROI3 > ROI4***
Group × stimulus	2, 111	1.772	0.175	
Group effect for target				
Group effect for standard				
Group × regions	6, 220	1.506	0.177	
Group effect at ROI1				
Group effect at ROI2				
Group effect at ROI3				
Group effect at ROI4				
Group × regions × stimulus	6, 220	0.864	0.522	

FEP, first-episode psychosis; CHR, clinical high risk; ROI, region of interest.

*** *P* < 0.001.

### Alpha oscillation

**Fig. 3:**
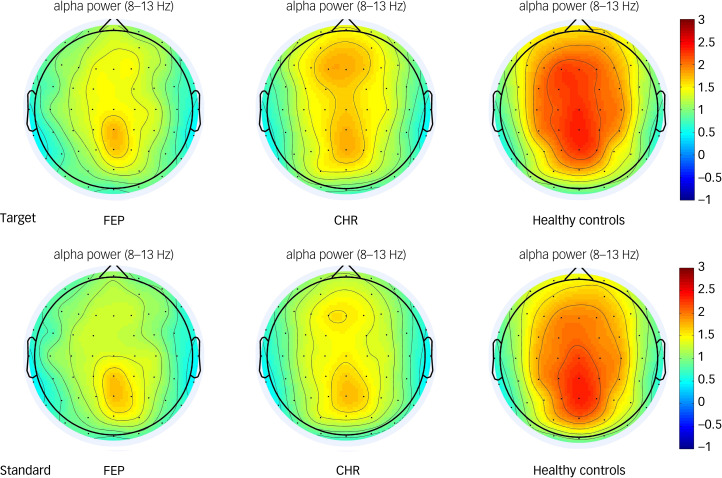
Topographic maps of the alpha oscillatory power of the target and standard stimulus in the first-episode psychosis (FEP), clinical high-risk (CHR) and healthy control groups. The colour bar represents the oscillatory power value.

Alpha oscillations were increased relative to baseline in the frontal, central, left fronto-central and right fronto-central areas in all three groups (Fig. 1). The group × region × stimulus interaction effect was not statistically significant (F(6,220) = 1.016, *P* = 0.416; as shown in Table 4). However, the main effect of brain region (ROI1, ROI2, ROI3, ROI4) was significant (F(3,109) = 20.582, *P* < 0.001). *Post hoc* tests showed that the alpha oscillation in the left fronto-central region was significantly larger than that in the right fronto-central region (*P* < 0.001), and that the alpha oscillation in the frontal region was significantly larger than that in central region (*P* < 0.001). The main effect of group was also significant. *Post hoc* tests showed that alpha oscillations in healthy controls were significantly higher than that in the FEP group (*P* < 0.01). Alpha oscillations in the CHR group were not significantly different from those in the healthy control and FEP groups (*P* > 0.05). Topographic maps of the alpha oscillations in the three groups to the target and standard stimuli are shown in Fig. 3.

**Table 4:** Effects of different factors on alpha oscillation

Effect	Hypothesis d.f., error d.f.	F/T	*P-*value	Follow-up tests
Group (FEP, CHR, healthy controls)	2, 111	6.061	0.003	Healthy controls > FEP**, healthy controls > CHR, CHR > FEP
Stimulus (target, standard)	1, 111	0.037	0.848	
Regions (ROI1, ROI2, ROI3, ROI4)	3, 109	20.582	<0.001	ROI3 > ROI4*** = ROI1 > ROI2***
Group × stimulus	2, 111	2.163	0.048	
Group effect for target	2, 111	3.994	0.021	Healthy controls > FEP*, healthy controls > CHR, CHR > FEP
Group effect for standard	2, 111	5.555	0.005	Healthy controls > FEP**, healthy controls > CHR, CHR > FEP
Group × regions	6, 220	0.806	0.449	
Group effect at ROI1				
Group effect at ROI2				
Group effect at ROI3				
Group effect at ROI4				
Group × regions × stimulus	6, 220	1.016	0.416	

FEP, first-episode psychosis; CHR, clinical high risk; ROI, region of interest.

*P < 0.05, **P < 0.01, ***P < 0.001.

## Discussion

In the present study, there was no difference in the amplitude of N2 in the frontal and parietal lobes between the patient and control groups. Previous studies of the characteristics of N2 amplitude in patients with psychosis have reported mixed results. For example, some studies suggest that deficits in the fronto-central N2 amplitude in patients with psychosis are related to specific deficits in neural functions related to recognising complex stimuli.^26,27^ In contrast, another study found that the N2 amplitude cannot be used to differentiate patients with FEP from controls.^28^ However, as a decreased N2 amplitude was found in patients with a chronic course of psychosis,^29^ it can be interpreted that a decreased N2 amplitude is related to the chronic course of the disease, and worsens with disease development. It is reasonable to assume that, during the early stages of psychosis, damage to N2-related neurons is minor, and the alterations in the time-domain amplitude reflected by ERP are insufficient to be observed. Our results also support the viewpoint that N2 amplitude is not a reliable biomarker of early-onset psychosis.^30^

This study did find that the theta-ERS after stimulus in the frontal, central, left fronto-central and right fronto-central regions in the FEP group was significantly smaller than that in healthy controls, which is consistent with previous research on adults with psychosis.^31^ In one such study, when the cognitive abilities required by the task increased, adult patients with psychosis did not show any load-dependent increase in theta oscillations in the fronto-posterior region.^32^ In a study using a working memory paradigm, healthy controls showed increased theta-ERS in the frontal central region as task difficulty increased, whereas adult patients with psychosis showed a decreased theta-ERS in the frontal lobe under all difficult task conditions.^33^ In the present study, we found impaired theta-ERS in adolescents with FEP. During simple tasks, the decreased theta oscillations is attributable to the advantages of automatic processing and the lower need for intrusive executive control functions; in contrast, the increased theta oscillations is attributable to the greater impact of interference as the task load increases, and the need for stronger executive control functions to suppress automatic processing and preparation dependent on expected responses.^32^ Thus, a possible explanation for decreased theta-ERS in adolescents with FEP is the absence of anticipatory guided action plans and the inability to monitor self-priming behaviours and predict upcoming events. However, theta oscillatory alterations in FEP are difficult to explain at present, because the neurobiological mechanisms behind low-frequency oscillations in psychosis are not as easily identified as those behind high-frequency oscillations, such as gamma oscillation.

Furthermore, alpha-ERS in the frontal, central, left fronto-central and right fronto-central regions in the FEP group were significantly smaller than that in the healthy controls. Alpha-ERS is thought to reflect active processing associated with memory maintenance or top-down inhibition functions.^34^ A significant reduction in alpha-ERS in the right parietal midline and bilateral occipital areas has been previously observed in patients with psychosis.^35^ Deficits in alpha oscillation in psychosis may be related to abnormal thalamic-cortical circuitry or cortical-cortical connectivity.^36^ Alternatively, it has been suggested that deficits in alpha oscillation in patients with psychosis reflect deficits in attention allocation and visual information processing.^18,37^ It should be noted that alpha-ERS in the left fronto-central region was significantly larger than that in the right fronto-central region in our study. A possible explanation for this result is the frontal alpha asymmetry. A recent study found that frontal alpha asymmetry was significantly lower in patients with psychosis than in healthy controls, suggesting relatively lower activation of left frontal electrodes.^38^ However, since all participants in this study were right-handed, it is unclear whether this reflects differences between the left and right hemispheres.

Although there were no significant differences in theta and alpha oscillations in the CHR group compared with healthy controls and the FEP group, there was a tendency for impaired neural oscillations in the CHR group compared with healthy controls, and impairment was not as significant as that in patients with FEP. The smaller sample size in the CHR group may explain the failure to detect significant nerve oscillation damage in this group. Nevertheless, this study provides the first evidence of potential alteration from low-frequency oscillations in CHR individuals. Since some individuals may show relevant structural and functional brain damage at the CHR stage of psychosis,^39^ follow-up of the prognosis of this population will be helpful for identifying biomarkers of the clinical stage of psychosis, clarifying the development of psychosis and developing appropriate treatment for those in need.

The following limitations of this study must be noted. First, we used a relatively simple oddball paradigm in this study to ensure that participants with severe psychotic symptoms could still complete the tasks. Second, as the sample size of this study was limited, the results should be interpreted with caution. Third, as this was a cross-sectional study, we cannot identify symptom development, disease outcomes or whether electroencephalogram results change with disease outcomes. Follow-up is needed to provide neurobiological evidence of whether neural oscillations can predict disease outcome.

In conclusion, having ruled out the effects of antipsychotic use and chronic disease duration, this study provides clear evidence of alterations in theta and alpha oscillations in frontal and central regions in children and adolescents with FEP, and a trend of related changes in patients with CHR. The early stages of psychosis is already characterised by functional abnormalities in the brain (abnormal neuroregulation mediated by N-methyl-D-aspartate receptors, for instance, is considered to be a key component in the pathophysiology of psychosis^40^). This study further confirms that abnormal rhythmic activity of neurons may constitute the pathophysiological mechanism of cognitive dysfunction related to early-onset psychosis.

## Data Availability

The supporting data for this study are accessible upon request from the corresponding author, X.C. However, please note that the data cannot be made publicly available due to privacy or ethical restrictions.
